# Implementation of Novel Affinity Ligand for Lentiviral Vector Purification

**DOI:** 10.3390/ijms24043354

**Published:** 2023-02-08

**Authors:** Ana Sofia Moreira, Sandra Bezemer, Tiago Q. Faria, Frank Detmers, Pim Hermans, Laurens Sierkstra, Ana Sofia Coroadinha, Cristina Peixoto

**Affiliations:** 1 IBET Instituto de Biologia Experimental e Tecnológica, Apartado 12, 2780-901 Oeiras, Portugal; 2 ITQB Instituto de Tecnologia Química e Biológica António Xavier, Universidade Nova de Lisboa, Avenida da República, 2780-157 Oeiras, Portugal; 3 Thermo Fisher Scientific, 2333 CH Leiden, The Netherlands

**Keywords:** viral vector manufacturing, affinity chromatography, cell therapy, gene therapy, lentiviral vector

## Abstract

The use of viral vectors as therapeutic products for multiple applications such as vaccines, cancer treatment, or gene therapies, has been growing exponentially. Therefore, improved manufacturing processes are needed to cope with the high number of functional particles required for clinical trials and, eventually, commercialization. Affinity chromatography (AC) can be used to simplify purification processes and generate clinical-grade products with high titer and purity. However, one of the major challenges in the purification of Lentiviral vectors (LVs) using AC is to combine a highly specific ligand with a gentle elution condition assuring the preservation of vector biological activity. In this work, we report for the first time the implementation of an AC resin to specifically purify VSV-G pseudotyped LVs. After ligand screening, different critical process parameters were assessed and optimized. A dynamic capacity of 1 × 10^11^ total particles per mL of resin was determined and an average recovery yield of 45% was found for the small-scale purification process. The established AC robustness was confirmed by the performance of an intermediate scale providing an infectious particles yield of 54%, which demonstrates the scalability and reproducibility of the AC matrix. Overall, this work contributes to increasing downstream process efficiency by delivering a purification technology that enables high purity, scalability, and process intensification in a single step, contributing to time-to-market reduction.

## 1. Introduction

Lentiviral vectors (LVs), a complex subclass of the *Retroviridae* family, have emerged as one of the most used delivering tools in cell and gene therapy [1]. The increasing interest in LVs is mostly related to their ability to transduce proliferating and non-proliferating cells [2], to integrate and deliver long-term gene expression [3], and to provide a safer integration profile when compared to γ-retroviral vectors [4]. This enveloped virus stands out as a versatile tool since it can be applied for the treatment of infectious diseases or genetic disorders [5,6], or even in the generation of chimeric antigen receptor (CAR)-T cells for cancer immunotherapies [7,8]. Of the several envelopes available, the G protein of vesicular stomatitis virus (VSV-G) is the most commonly used, either in development research or in clinical applications, since it presents broad tropism and relatively high stability [9,10]. The replacement of the native envelope protein with a clinically relevant glycoprotein, pseudotyping, is another attribute of the LVs. Remarkably, from the list of more than 20 cell and gene products already on the market, 7 are LV-based therapies demonstrating the success of this viral vector [11]. Even though there is no strict value for therapeutic doses for either ex vivo or in vivo applications, most authors have been reporting viral doses between 10^10^ and 10^12^ per patient [12,13]. The growing interest in LVs exposes the current bottlenecks underlying downstream processing (DSP). LVs are known for their biological complexity, since viral stability can be affected by temperature [14,15], ionic strength [16,17], pH [14], freeze-and-thaw cycles [14,18], and shear stress [19]. Despite the significant improvements that have been made over the past few years, the production and purification of LVs are far from reaching their maximum potential. Currently, manufacturing platforms cannot fulfill clinical demands, reinforcing the need for robust, scalable, and cost-effective processes to sustain the commercialization of new LV-based therapies [20,21,22]. The downstream process of LVs comprises several operation units where the primary goal consists of concentration and purification of the virus, while preserving the biological activity [17,19]. Viral capture is probably one of the most challenging operation steps in the LVs purification process and, for that reason, different chromatography modalities have been investigated as alternatives to overcome low recovery yields [16,17,23,24]. Affinity chromatography (AC) explores highly selective and reversible interactions between the immobilized ligand and the product of interest, offering high purity, high fold concentration, and scalability in a single step [25,26,27]. This robust modality can generate economic benefits since it increases process standardization and simplification by reducing the number of unit operations [28]. Therefore, different approaches are described in the literature for the purification of LVs using affinity chromatography. Immobilized metal affinity chromatography (IMAC), involving envelope protein engineering with a histidine tag (his-tag), presents a low-cost and highly stable solution [29]. However, concerns regarding the harsh desorption agents resulting in viral inactivation (e.g., imidazole or EDTA) or the possible adverse effects in further clinical applications (e.g., proteases, his-tag ligands, metal ions leakage) have been a major drawback [19,30]. Another envelope affinity tag strategy was the expression of biotin onto the LVs surface for capture with immobilized streptavidin [31,32]. Recently, the purification of an RDPro pseudotyped LV labeled with cTag8 was also described by Mekkaoui et al. where a biotin mimic was genetically encoded and pseudotype-independent [33]. Additionally, heparin affinity can be an attractive technology as it presents affordable costs and an elution step at mild conditions, contributing to viral stability [1]. Nevertheless, the lack of selectivity leads to DNA and host cell proteins (HCP) co-elution. Furthermore, most commercial heparin ligands have animal origin, which brings some constraints for clinical applications, since the traceability and validation of raw materials can be challenging [16,34,35]. Therefore, this highlights the need for an affinity chromatography step that can offer high specificity and scalability using gentle elution conditions compatible with the biological activity of the enveloped viral vectors. Over recent years, the use of recombinant camelid-derived single-domain antibody fragments (V_H_H) has been reported for several biopharmaceuticals [36], such as different AAV serotypes [27,37]; more recently, Moleirinho (2020) et al. used similar technology to develop a negative mode AC for baculovirus removal [38]. Due to its small size, high specificity, mild elution conditions, and free from animal components, V_H_H ligands can be an alternative to mitigate some of the concerns in affinity approaches.

In this work, the first affinity matrix design to bind specifically to VSV-G pseudotyped LVs was successfully established. Initially, a set of ligands produced by phage display was screened for viral specificity and elution efficiency in mild conditions. The lead candidates were evaluated using functional assays to determine the best performer. In addition, the critical process parameters were assessed and optimized for the lead candidate. Afterward, the full performance of the established Lenti VSVG Affinity Matrix was determined at different scales. Finally, LVs purified with the novel affinity matrix were characterized in terms of impurity removal and quality attributes.

## 2. Results and Discussion

### 2.1. Ligand Discover and Initial Scouting

To generate novel affinity ligands that could specifically bind VSV-G pseudotyped LVs (VSVG-LVs), a non-immune V_H_H library was used to specifically enrich VSVG-LVs binders by phage display. After isolation of single clones, a set of V_H_H ligands showing binding to VSVG-LVs particles in a surface plasmon resonance (SPR) array was selected for further analysis and re-cloned into a yeast production strain. Based on yeast expression results, a set containing lead candidates was composed for the reactivity analysis.

#### 2.1.1. Binding Reactivity Using Biotin–Ligand Conjugates

**Figure 1:**
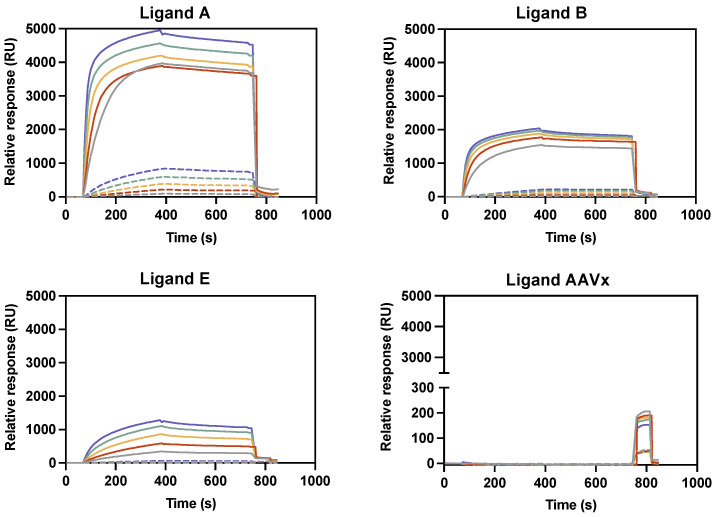
LVs binding reactivity using biotin–ligand conjugates. Representation of the SPR sensorgrams containing the relative response (RU) versus time (s). The three different interaction profiles observed between the VSVG-LVs and the novel ligands are illustrated by the ligand A, B, E and by the negative control with the AAVX ligand. For each ligand, different binding responses were obtained by varying the dilution of a VSVG-LVs feedstock injected (2-fold dilutions from 10 to 160 times). Color code: blue represents 10 times dilution, green represents 20 times dilution, yellow represents 40 times dilution, red represents 80 times dilution, and gray represents 160 times dilution. Solid lines represent the experiments performed using the maximum biotinylated V_H_H fragments concentration (20 μg/mL) spotted onto a Strep G Senseye biosensor chip, whereas dashed lines represent the minimum concentration used (0.02 μg/mL).

The binding reactivity of the yeast produced V_H_H ligands was initially screened by surface plasmon resonance (SPR) array. To study the relative response (RU) of the anti-VSVG-LVs V_H_H ligands (ligands A to E), different concentrations of VSVG-LVs were injected in the sensors at four concentrations of each ligand (Figure 1 and Figures S1–S5). Additionally, two negative controls, BacuClear and AAVX affinity ligands with specificity for baculovirus and adeno-associated virus respectively, were included in the SPR assay (Figures S6 and S7). The sensorgrams depicted in Figure 1 contain examples of the three different interaction profiles observed between the VSVG-LVs and the novel ligands. All the remaining binding profiles are represented in Figures S1–S7. In this preliminary screen, the highest binding reactivity was found for the ligands A and D with a value of 5000 RU, followed by the ligands B and C, which displayed values around 2000 RU. Regarding ligand E, a moderate binding response was observed, which was four times lower when compared with the highest response obtained. For BacuClear and AAVX ligands, no reactivity towards VSVG-LVs particles was observed, supporting the apparent specificity of the generated ligands. According to the SPR results, it was possible to obtain an indication of the most promising candidates that were further evaluated using functional assays.

#### 2.1.2. VSVG-LVs Release Efficiency Using Biotin–Ligand Conjugates

**Figure 2:**
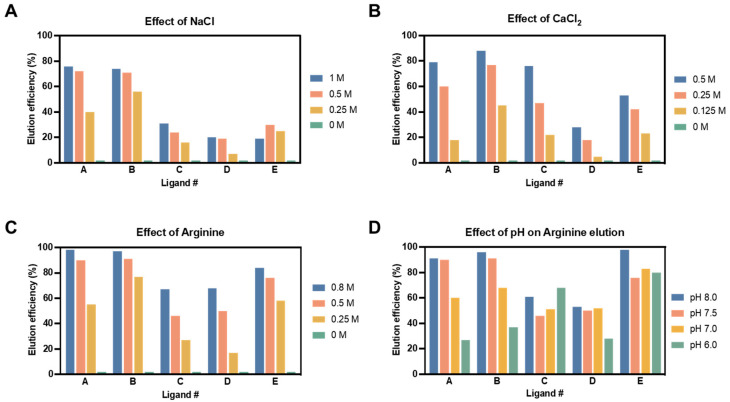
LVs release efficiency using biotin–ligand conjugates. Impact of different desorption buffers, sodium chloride (**A**), calcium chloride (**B**), and arginine (**C**), on elution efficiency (%) for the ligand candidates assessed by SPR assay. Three concentrations were included in the buffer elution optimization. (**D**) The influence of the elution pH on elution efficiency (%) was investigated using an intermediate arginine concentration, 0.5 M, at four pH values (8.0, 7.5, 7.0, and 6.0).

Although ligands should present high affinity towards the viral target, a reversible binding at mild conditions is necessary to establish an efficient purification step for this envelope virus. Thus, the ligand scouting was focused not only on specificity but also on elution efficiency. Different elution buffers were evaluated in this work, such as monovalent (sodium chloride) and divalent (calcium chloride) salts and also arginine. Even though the first ones are relatively common, arginine has been used for different applications, such as suppressing protein aggregation [39], promoting protein refolding [40], as a stabilizing agent [41], and has also been reported to improve milder elution of antibodies from protein-A affinity resins [42,43,44]. In these experiments, three concentrations for each elution solvent were evaluated using 50 mM Tris as a buffer. This initial assessment was performed at pH 7.5 to avoid viral inactivation. Except for ligand E, in all the remaining ligands, elution efficiency was improved by increasing the elution buffer concentration (Figure 2A–C). The results obtained suggested that release efficiency was ligand-dependent when using sodium chloride (Figure 2A). For this elution buffer, the highest viral release was obtained for ligands A and B with 1 M of salt, where the maximum value of 76% was achieved for ligand A. However, the same behavior was not observed in ligands C, D, and E, where elution efficiency was lower than 30% for all conditions. Moreover, the high salt concentration necessary to achieve high elution release was considered a major drawback since it can lead to viral inactivation [45]. Thus, sodium chloride was excluded as an elution buffer in this study. On the other side, calcium chloride (Figure 2B) and arginine (Figure 2C) presented similar tendencies regarding the viral release. Accordingly, since the elution efficiencies obtained for the arginine buffer were higher for all ligands when compared with the calcium chloride, the first one was chosen as the final elution agent. The presence of several charged groups in its structure (positively charged amines and negatively charged C-terminal carboxylic acid) could be the explanation for the good performance of arginine across the different affinity ligands [46]. This strategy has also been applied to other viral vectors; for instance, in the purification of adeno-associated viruses [47]. The effect of the pH on viral release was also investigated for the selected buffer. In this case, the elution efficiency was evaluated using the intermediate arginine concentration at four pH values. Figure 2D depicts the variation in viral release across the ligand candidates. The results obtained showed that elution efficiency was higher at pH 8 for all ligands except for ligand C, where the highest elution value was achieved at pH 6; curiously, the pH value at which worst elution was observed for the other ligands. LVs are sensitive to extreme pH values, being its negative impact extensively studied and reported by different authors [14,48]. Therefore, the selection rationale was to find a compromise between elution efficiency and viral compatibility [18], and so pH 7.5 was the condition selected to proceed for further studies.

### 2.2. Generation of VSVG-LVs Affinity Chromatography Prototype

#### 2.2.1. VSVG-LVs Depletion Using Streptavidin Agarose Beads

**Figure 3:**
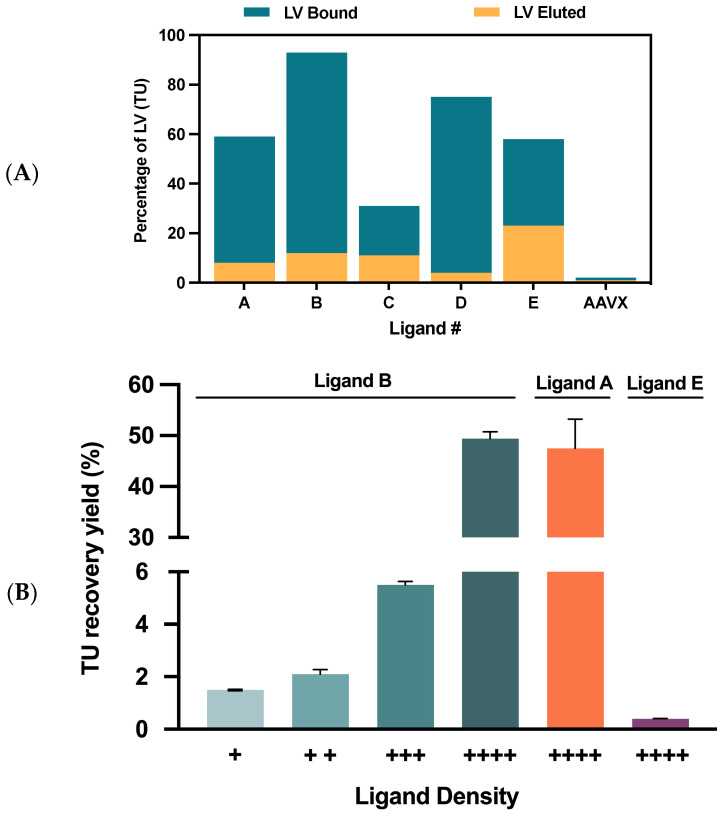
Lead affinity ligand candidate selection. (**A**) Adsorber screening using streptavidin agarose beads functionalized with biotinylated ligands (A to E). Transducing units’ depletion (blue bars) and elution (yellow bars) comparison between the different ligands. The AAVX ligand was included as a negative control. (**B**) Transduction units’ recovery comparison between A, B, and E adsorber prototypes at the highest ligand density (++++). The effect of ligand density was evaluated for ligand B as it was shown to be the leading candidate. Ligand densities are ordered from the lowest (+) to the highest (++++) value evaluated. Color code: the four bars colored with shades of green correspond to different ligand densities of ligand A, orange correspond to ligand B and purple bar to ligand E.

To assess VSVG-LVs’ functional recovery yield in a small-scale model, streptavidin agarose beads were functionalized with biotinylated ligands selected by the SPR assays. This model was a versatile tool as it offered stable immobilization chemistry and high binding capacity. Thus, VSVG-LVs’ bound—or viral depletion—was estimated by comparing the functional particle’s titer in the supernatant before and after capture. Results showed that maximum depletion was observed for ligands B and D with more than 75% viral depletion, which confirms the high binding capacity of these ligands. Regarding viral elution, all the ligands presented values below 23%, ligand E being the best performer (Figure 3A). The low elution yields observed can be explained by the small-scale setting (50 µL beads) used, and by the fact that the values obtained were near the limit of detection of the FACS equipment. The AAVX resin was also included in this experiment as a negative control with no binding of VSVG-LVs particles being observed, thus reinforcing the specificity of the ligands evaluated. The final three leading candidates were selected based on two criteria: ligand depletion capacity and elution efficiency. Although ligand D presented a viral depletion of 75%, it showed a poor elution yield, lower than 4%. This may be caused by a too-strong interaction between the ligand and the viral vector preventing elution at mild conditions [26]. For this reason, ligands A, B, and E were chosen for further evaluation.

#### 2.2.2. Matrix and Final Ligand Evaluation

After selecting the final candidates, it was necessary to decide on an appropriate matrix to assemble the novel affinity prototypes. To be considered an ideal matrix, a few requisites should be met, such as homogeneity, stability, residual nonspecific binding, large surface area for ligand attachment, and easy ligand coupling [25,49]. Therefore, to determine the most suitable resin for functionalization with the VSVG-LVs binding ligands, a set of different matrices were compared (Table 1). Except for matrix 4, all the other media presented similar particle size and the same coupling method. Results suggest that matrix 3 was the best performer, showing the higher selectivity profile and consequently minimal nonspecific binding towards the VSVG-LVs particles. Once the matrix was chosen, it was possible to produce adsorber prototypes with the final three ligands. To validate the depletion results obtained using the streptavidin agarose beads, VSVG-LVs capture was determined using packed columns at a 1 mL scale. Since ligand B presented the highest viral depletion, four ligand densities were investigated for this prototype. Regarding the remaining candidates, viral depletion was studied only in the highest ligand density condition (Figure 3B). Both ligands A and B achieved near 50% recovery yield in the maximum ligand density, with the latter ligand performing slightly better. On the opposite side, ligand E’s performance did not corroborate the previous results, showing a residual recovery yield of approximately 1%. Considering all these results, ligand B at the highest ligand density was selected to produce the novel affinity resin prototype that will be referred to as Capture Select Lenti VSVG Affinity Matrix.

### 2.3. Chromatography Optimization for the Lead Candidate

An affinity chromatography media should offer several prerequisites related to its performance, such as high binding capacity, high recovery yield, maintenance of product biological activity, high removal of impurities, inherent in each system [50]. To discover the full potential of the assembled new matrix and define the best operating conditions, some critical process parameters (CPP) were explored and optimized.

#### 2.3.1. Residence Time Evaluation

**Figure 4:**
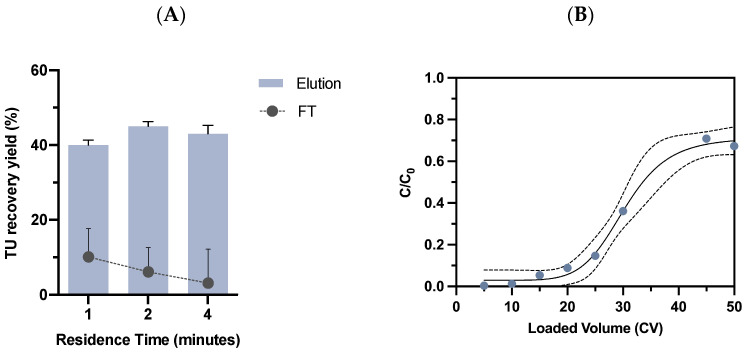
Process parameter optimization for the novel affinity adsorber. (**A**) Comparative VSVG-LVs recovery yield results for different residence times (1, 2, and 4 min). For each condition, the viral recovery and viral loss was estimated. (**B**) Breakthrough curve representation for the Lenti VSVG Affinity Matrix. The clarified VSVG-LVs were loaded at flow velocity of 72 cm/h in a 1 mL packed bed. C is the virus total particles’ concentration in the flow-through samples and C_0_ is the virus concentration of feedstock material, the C_0_/C ratio is represented by blue dots. Dashed lines represent the 95% confidence interval. Total particle concentration was estimated by detection of p24 protein.

Residence time (RT) usually plays an important role in affinity resins’ performance. The optimization of this process parameter can lead to the increase of the dynamic binding capacity and, consequently, process efficiency improvements. In order to assess the influence of residence time on the Lenti VSVG Affinity Matrix, clarified VSVG-LVs were loaded at a linear velocity of 144 cm/h, 72 cm/h, and 36 cm/h, corresponding to residence times of 1, 2, and 4 min, respectively. Regarding viral capture, the residence time of 1 min was the worst condition, resulting in the loss of 10% in the flow-through fraction combined with the lowest TU recovery yield of 40% after elution (Figure 4A). As expected, the performance of the adsorber improved by increasing the residence time with a maximum recovery of 45% at a residence time of 2 min, while the lowest viral loss in the flow-through was observed for the residence time of 4 min. However, at these residence times, no meaningful differences were observed, indicating a similar performance of the adsorber for these conditions. In fact, these results are in line with previous reports where the impact of the residence times on resins’ productivity has been studied. Several authors have demonstrated that shorter residence times can result in reduced resin capacity [50,51,52]. To select the most suitable residence time, another parameter must be taken into consideration as it can influence the success of LVs manufacturing: the processing time. One of the most challenging features of this enveloped virus is its short half-life of 5–8 h at 37 °C [14,53,54]. This highlights the need to reduce the time of the production process and establish fast, efficient, and simpler purification technologies [1,55]. Therefore, the 2 min residence time was selected, aiming to contribute to process time reduction. Notably, the present adsorber exhibited a competitive performance when compared with existing affinity platforms for viral capture. Most affinity technologies commercially available have been reporting a contact time of 0.5–3 min, such as POROS™ CaptureSelect™ BacuClear, AAVX, AAV8, and AAV9 [20,38,56,57].

#### 2.3.2. Dynamic Binding Capacity Determination

After determining the residence time that maximized viral recovery and minimized viral loss, the dynamic binding capacity (DBC) of the Lenti VSVG Affinity Matrix was evaluated. A breakthrough curve for the novel matrix was built using a 1 mL packed column, loaded with 50 CV of clarified harvest at 4.0 × 10^9^ TP/mL (Figure 4B). Samples at different loaded volumes were collected to determine the total particle concentration. A DBC_10%_ of approximately 1.0 × 10^11^ TP/mL of resin was determined from the breakthrough curve that displayed a typical sigmoid shape. When compared with the 1.3 × 10^10^ TP/mL binding capacity reported by Mercedes (2005) et al. [16] for the well-known Heparin affinity resin, the Lenti VSVG Affinity Matrix adsorber achieved a 10-fold improvement, which can have a positive impact in terms of process scalability. Considering other affinity resins based on CaptureSelect™ technology, it can be stated that the performance achieved is in line with that of the existing adsorbers. The superior binding capacity found for the different AAV serotypes resins, which ranged from 10^13^ to 10^14^ genome containing particles/mL of resin [37], may be related to the viral particle size. The size of an AAV vector is approximately 20 nm, whereas for LVs it is six times larger. This can lead to a reduction in the surface area available and result in lower binding capacities [28]. Undeniably, different parameters can influence the capacity of affinity supports. Not only by resin physical properties (e.g., bead and pore size, ligand density, and conformation, surface chemistry) but also by the critical process parameters (e.g., pH, buffer system, temperature, linear flow rate, particle size) [58,59,60]. Additionally, all the inherent impurities associated with each system, for instance, the presence of free VSV-G proteins, can behave as competitors for the affinity ligand and impact resin capacity.

#### 2.3.3. Affinity Chromatography Performance and Scalability Evaluation

Considering all the previous results, the performance of the VSVG-LVs affinity matrix was assessed. A 1 mL packed column was used to load 25 CV of VSVG-LVs containing supernatant with a residence time of 2 min, and viral elution was performed in a single isocratic step of 0.8 M arginine at pH 7.5.

**Figure 5:**
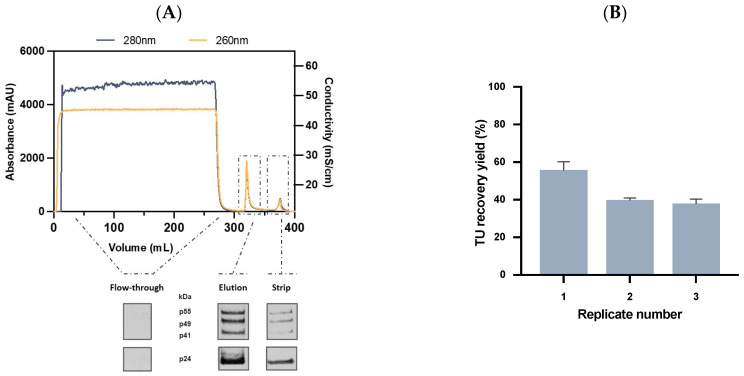
Evaluation of a novel Affinity chromatography for lentiviral vector purification. (**A**) Chromatogram of VSVG-LVs purification using Lenti VSVG Affinity Matrix at 1 mL scale and 2 min residence time (top) and Western blot signal profile for flow-through, elution, and strip fractions using anti-p24 antibody (bottom). Blue and yellow lines represent absorbance at 280 nm and 260 nm, respectively. (**B**) Purification performance of the VSVG affinity resin at a 1 mL scale. TU recovery yield (%) is represented for each replicate (*n* = 3). Error bars are the standard deviation obtained from the titration method.

To determine the LVs recovery yield, the initial sample, flow-through, and elution fractions were collected and analyzed for total particle concentration and transduction efficiency. Column striping was also collected and analyzed. A standard chromatographic profile for the LVs’ purification using the novel affinity adsorber is shown in Figure 5A. On the bottom is represented the Western blot analysis using the Gag p24 protein antibody in all process samples. In the flow-through, no signal was detected showing that the Lenti VSVG Affinity Matrix could successfully capture the VSVG-LVs particles without resin saturation. In contrast, a strong signal was found in the elution fraction, which exhibited the characteristic LVs p24 pattern [61,62], supporting that an efficient viral elution occurred. For the strip sample, a subtle signal was detected, although it was stronger for the p24 protein. This can indicate that only p24 free protein was eluted after column striping and a viral quality improvement is expected. To verify the reproducibility of the affinity adsorber, three similar runs at 1 mL scale were performed. Clarified VSVG-LVs at an average concentration of 5.1 ± 2.5 × 10^7^ TU/mL were loaded in a total of 25 mL, and the results are presented in Figure 5B. The elution step retrieved an average of 45 ± 10% of the loaded virus, while 6 ± 3% were lost in the flow-through. These results show the reproducibility and high elution efficiency of this adsorber. Importantly, viral activity was retained, demonstrating good compatibility between LVs and mild elution conditions. Additionally, in just one purification step a volumetric concentration factor (VCF) of 6 was achieved together with a 3-fold increase in the TU concentration. After a successful purification at a small scale, it was necessary to evaluate the scalability of the novel affinity resin. Therefore, an intermediate scale-up of 10 mL resin was performed. In this case, 250 mL of clarified VSVG-LVs at an average concentration of 7.3 ± 2.1 × 10^7^ TU/mL (Table 2) were loaded, and the linear velocity was adjusted to 150 cm/h in order to maintain the residence time at 2 min.

**Figure 6:**
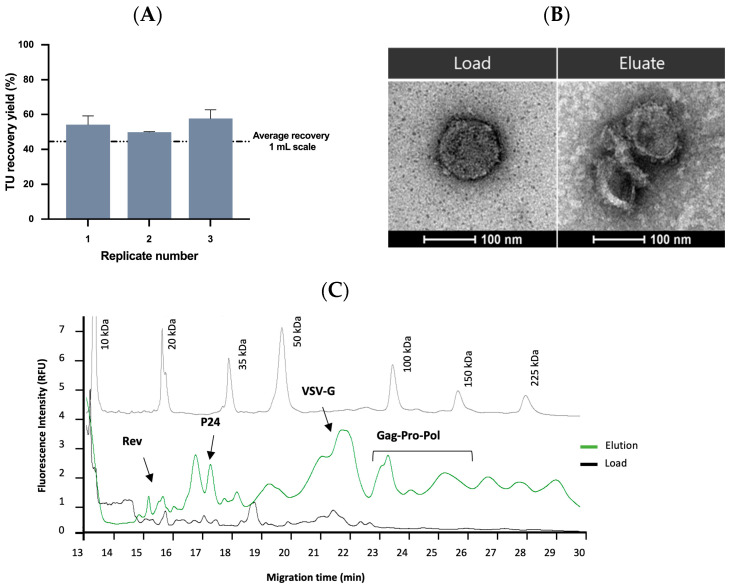
Intermediate scale-up and characterization of purified VSVG-LVs. (**A**) VSVG-LVs purification at 10 mL scale. The graphic represents the TU yield (%) for each replicate (*n* = 3). Dashed line shows the average recovery yield obtained from 1 mL scale (45 ± 10%). Error bars are the standard deviation obtained from the titration method. (**B**) TEM analysis of VSVG-LVs initial sample (left image) and after purification with the Lenti VSVG Affinity Matrix (right image). Scale bar 100 nm. (**C**) Electropherograms represent the molecular weight marker (gray line), the clarified VSVG-LVs sample (black line), and VSVG-LVs purified with the Lenti VSVG Affinity Matrix (green line) using CE-SDS with a LIF detector. Major VSVG-LVs proteins are indicated.

The results obtained showed a slight improvement in comparison with the 1 mL scale by displaying an average recovery yield of 54 ± 4%, and only 4 ± 4% was lost in the flow-through (Figure 6A). A 5-fold increase in TU concentration and a VCF of 12 were observed. These results reinforce the high selectivity, reproducibility, and scalability of this new affinity resin. Over recent decades, different LVs purification methodologies have been reported from AC to AEX. For instance, Mercedes et al. showed a recovery yield of 53%, although resin scalability was not addressed [16,35]. The LVs’ purification using Mustang Q membrane has also been studied, where a cumulative recovery yield of 90% was demonstrated by performing the elution with two NaCl steps [23]. However, LVs eluted in each step presented different purity profiles being the virus eluted with 0.4 M NaCl (comprising 37% of VSVG-LVs) more contaminated with residual proteins and fractions eluted with 1.5 M NaCl (containing 56% of the viral vectors) contaminated with DNA. Additionally, due to the high salt concentration used for viral elution, a dilution step was necessary for viral stability and, eventually, followed by a final concentration and formulation step. Other chromatographic media have also been explored for LVs’ capture; for instance, monoliths or even nanofibers [17,63]. Recently, the use of steric exclusion using hydrophilic cellulose membranes for the purification of these viruses was reported. Although interesting results have been achieved, a deeper study is necessary to enable the scale-up of these devices. Overall, when compared to some of these technologies, the novel affinity resin showed a competitive performance.

### 2.4. Quality Control and Vector Characterization

Currently, the number of clinical trials using LVs for regulatory approval is increasing. As a result, clinical production at large-scale, according to current good manufacturing practice (cGMP), is becoming more stringent, thus requiring an extensive vector characterization for batch release. Consequently, analytical methods have a decisive role during process development, since it has been shown that the impurity level can impact the transducibility of γ-retroviral vectors and LVs [55,64]. Affinity chromatography allows process simplification, high recovery yields, and high purity in the final product. To characterize the purity level of purified VSVG-LVs using the novel affinity resin, different analytical techniques were used. A total protein removal higher than 92% and a total DNA removal higher than 80% were observed after the chromatographic step, most of these impurities being washed out in the flow-through samples. Moreover, the high impurity clearance observed is in agreement with results obtained for different technologies in the LVs purification field [23,55,65]. For some cases, the total DNA removal attained in this work was even superior [35,66]. Another important quality attribute is the capacity of removing host cell proteins (HCP) derived from the producer cell line (HEK293T). An overall clearance of 99% was achieved, obtaining a total of 1.5 ± 0.2 µg of HCP for a viral dose of 10^9^ TU. The novel affinity resin was able to match (e.g., 0.484–1.631 µg of HCP/10^9^ TU) or even decrease (e.g., 5.9 ± 0.2 µg of HCP/10^9^ TU) HCP content in a single step, when compared to similar viral doses obtained after a full purification process [23,65]. In addition to impurity clearance, viral quality in terms of the ratio between total particle and transducing units (TP/TU) is also relevant. In Table 2, a summary of TP/TU ratio progress during the purification process is presented. The results indicate a quality improvement by the reduction of the TP/TU ratio after the elution step. Although no reference value was established in current guidelines, most clinical grade LVs reported higher values around 10^3^ [22,67]. Afterward, viral integrity was investigated by transmission electron microscopy (TEM). TEM analysis confirmed the presence of LV particles on the initial and elution fractions (Figure 6B); both presented a vector size slightly larger than 100 nm. Comparing both images, it was observed that shape and morphology were maintained after the purification step using the Lenti VSVG Affinity Matrix. In fact, in the eluate image it was also possible to discriminate the envelope proteins displayed on the virus surface. Undeniably, these results corroborate the good compatibility between LVs particles and the gentle nature of the established elution step. Lastly, capillary electrophoresis (CE) is a promising technique that has demonstrated its potential in the viral analytical field. Figure 6C illustrates the comparison between the obtained electropherograms of the clarified and affinity purified VSVG-LVs. CE-SDS LIF analysis was used to assess the protein profile of the purified VSVG-LVs and it was possible to identify four LV proteins: Rev (19 kDa), p24 (24 kDa), Gag-Pol-Pro (80–160 kDa), and the envelope protein VSV-G (58 kDa) [68,69].

## 3. Materials and Methods

### 3.1. Lentiviral Vector Production

#### 3.1.1. Cell Line Maintenance and Transfection

HEK293T cells (ATCC^®^ CRL-3216™) obtained from the American Type Culture Collection (ATCC, Manassas, VA, USA) were adapted to grow in suspension as described in [70]. The cells were cultivated in BalanCD^®^ HEK293 medium (FUJIFILM IrvineScientific, Santa Ana, CA, USA) supplemented with 4 mM GlutaMAX™ (Thermo Fisher Scientific™, Waltham, MA, USA) and maintained at 37 °C in an incubator with a humidified atmosphere of 8% CO_2_ in the air. Cell concentration and viability were assessed by the trypan blue exclusion method. LVs were produced by transient transfection using pALD-Lenti System (Aldevron^®^, Fargo, ND, USA) including the pALD-VSV-G-K, pALD-GagPol-K, pALD-Rev-K, and pALD-Lenti-EGFP-K plasmids. Briefly, the cells were transfected at 2.5 × 10^6^ cells/mL in a shake flask using linear 25 kDa polyethyleneimine, PEIpro^®^ Transfection Reagent (Polyplus-transfection^®^, Illkirch-Graffenstaden, France) at a mass ratio of 1:3 (DNA:PEI) and 1 µg of total DNA per 1 × 10^6^ cells. At 48 h-*post*-transfection, VSVG-pseudotyped LVs (VSVG-LVs) were harvested at a cell density ranging from 4–6 × 10^6^ cells/mL. 

#### 3.1.2. Nuclease Treatment and Virus Clarification

Nucleic acid digestion was performed with Benzonase^®^ endonuclease (Merck, Darmstadt, Germany) at a final concentration of 100 U/mL and in the presence of 2 mM MgCl_2_. The digestion proceeded for 30 min at room temperature. The VSVG-LVs containing supernatant were clarified by centrifugation at 500× *g* for 10 min at 18 °C followed by filtration using a Nalgene™ Rapid-Flow™ Sterile Single Use Vacuum Filter Unit with a PES (Polyethersulfone) membrane with 0.45 µm pore size (Thermo Fisher Scientific™, Waltham, MA, USA).

### 3.2. Discovery, Selection, and Production of V_H_H Fragments

Phage display was performed using Thermo Fisher’s non-immune V_H_H libraries following a screening of V_H_H supernatants derived from isolated single clones in a label-free binding assay based on surface plasmon resonance (SPR) as described by Moleirinho (2020) et al. [38]. In short, Maxisorp^®^ flat-bottom, 96-well plates (Thermo Fisher Scientific™, Waltham, MA, USA) were pre-coated overnight at 4 °C with a 10 times dilution of a VSVG-LVs preparation in PBS, pH 7.4 at 100 μL/well at a final estimated concentration of ~1 × 10^11^ total particles per mL. The plates were washed, and then subsequently blocked for 1 h at room temperature with 2% *w*/*v* dried skimmed milk (Protifar, Dublin, Ireland) in PBS at 200 μL/well (2% P-PBS). V_H_H phages from the non-immune libraries were diluted 10 times in 1% PBS, 0.05% (*v*/*v*) Tween-20 (1% PBST) and incubated for 1 h at room temperature on the plates. After vigorous washing of the wells with PBST, the residually bound V_H_H phages were eluted following a rescue of the phagemids by infection of TG1 cells as described by Adams (2014) et al. [71]. A second round of phage display was performed with the enriched libraries, now using a 10—and 25 times dilution of the V_H_H phages. After the second round of selection, single TG1 clones from the VSVG-LVs enriched libraries were isolated following V_H_H production in deep well microtiter plates. The crude *E. coli* supernatants with soluble V_H_H fragments equipped with a C-terminal tag sequence amenable to site-specific enzymatic biotinylation [72] were directly spotted onto a streptavidin biosensor chip (Strep G Senseye, Enschede, The Netherlands) using a continuous flow microspotter (Carterra^®^, Salt Lake City, UT, USA) according to the manufacturer’s protocol. The biosensor chip, now functionalized with an array of V_H_H fragments, was used in a first screen to identify binders against VSVG-LVs particles using the IBIS MX96 instrument (IBIS technologies, Enschede, The Netherlands) that facilitates label-free and real-time binding analysis based on SPR. The sequences encoding V_H_Hs of the positive clones identified in this first screen were elucidated by Sanger sequencing (BaseClear, Leiden, The Netherlands). In order to obtain sufficient V_H_H material for further characterization of the positive binders in a second screen, the unique V_H_H sequences were re-cloned into a yeast expression vector following the integration of the V_H_H genes into the genome of *Saccharomyces cerevisiae* as previously described [36,73]. V_H_H production was carried out at shake-flask scale followed by purification from the extracellular medium using cation exchange chromatography. The purified V_H_H fragments were chemically conjugated to biotin by Thermo Fisher Scientific according to their standard procedures. The resulting biotin conjugates facilitated more detailed characterization of the V_H_H fragments on functionality and selectivity in SPR, and streptavidin agarose beads-based assay setups.

### 3.3. Surface Plasmon Resonance (SPR)

#### 3.3.1. Binding Profiles

The purified and chemically biotinylated V_H_H fragments were diluted in PBST at different concentrations (i.e., 20, 2, 0.2, and 0.02 μg/mL) and subsequently spotted onto a Strep G Senseye biosensor chip as previously described. To determine potential non-specific binding of the VSVG-LVs particles to the biosensor surface, two irrelevant V_H_H fragments directed against AAV and baculovirus (Thermo Fisher Scientific™, Waltham, MA, USA) were included as biotin conjugate using the same concentration series for spotting. The functionalized biosensor was placed into the IBIS MX96 instrument following a standard run for target binding analysis using PBST as running buffer. Binding profiles of the different V_H_H fragments were analyzed using the VSVG-LVs preparation diluted 10, 20, 40, 80, and 160 times in running buffer from an initial sample at an estimated concentration of ~1 × 10^11^ total particles per mL. Briefly, after setting a baseline with running buffer for 1 min, the samples were allowed to bind to the spotted array of V_H_H fragments for 5 min (association phase) following a washing step with running buffer for 5 min (dissociation phase). After each run, the biosensor was regenerated using 100 mM tri-ethylamine (TEA) for 1 min following a re-equilibration step with running buffer for 30 s. For each spotted V_H_H fragment of the SPR-array, individual sensorgrams were generated per sample and an overlay of all resulting sensorgrams per spot was obtained using the IBIS MX96 software.

#### 3.3.2. Elution Efficiency

To study the release of the bound VSVG-LVs particles under mild elution conditions by SPR, a variety of elution buffers was evaluated using 50 mM Tris, pH 7.5 as buffer system. The elution buffers used were either arginine up to a level of 800 mM, NaCl or CaCl_2_ up to a level of 1 M and 0.5 M, respectively. For the arginine buffer, different pH values were tested ranging from pH 6.0 to pH 8.0. For target binding, the VSVG-LVs preparation was diluted 80 times in 50 mM Tris, 150 mM NaCl, 0.075% (*v*/*v*) Tween-20, pH 7 (running buffer). After binding, the target release efficiency of each elution buffer composition was determined. In short, after setting a baseline with running buffer for 1 min, the VSVG-LVs sample was allowed to bind to the spotted array for 5 min (association phase) following a wash step with running buffer for 1 min (endpoint set as response signal before elution). After the wash step, an elution buffer composition was incubated for 5 min following a wash step with running buffer for 1 min (endpoint set as response signal after elution). After each run, the biosensor was regenerated using TEA for 1 min following a re-equilibration step with running buffer for 30 s. The difference in response signal before and after the elution step was determined to calculate the target release efficiency for each of the buffer compositions tested using the IBIS MX96 software. If the response signal after the elution step reached baseline, the elution efficiency was valued 100%, whereas in the case that no decrease was observed, the elution efficiency was valued at 0%.

### 3.4. VSVG-LVs Viral Capture Using Streptavidin Agarose Beads

Selected biotinylated VSVG-LVs binding V_H_H fragments were coupled to high-capacity streptavidin agarose beads (Thermo Fisher Scientific™, Waltham, MA, USA) following the manufacturer’s instructions. Beads were washed and equilibrated using 50 mM HEPES, 150 mM NaCl at pH 7.5 as binding buffer. Briefly, the produced VSVG-LVs were incubated for 1 h with the 50 µL streptavidin agarose beads under gentle agitation at room temperature. Afterward, the supernatant was collected by centrifugation, followed by washing the beads three times with binding buffer. Viral elution was performed with 50 mM HEPES, 150 mM NaCl, 800 mM arginine at pH 7.5, and the strip step was performed by boiling the beads in SDS-PAGE sample buffer. The collected samples (flow-through, elution, and strip) were stored at −80 °C for further analysis.

### 3.5. Generation of Prototype Affinity Resins

In order to obtain sufficient V_H_H material for preparing prototype affinity resins to evaluate performance in chromatography, the selected VSVG-LVs binding V_H_H fragments were produced at larger scale by running 10 L yeast fermentations following a 2-step purification protocol using ion-exchange chromatography (IEC). This setup, carried out by Thermo Fisher Scientific, also verifies whether the selected V_H_H candidates can be manufactured at an economically feasible scale. The purified V_H_H fragments that passed all scalability requirements were chemically coupled to different types of resin beads and at different V_H_H (ligand) densities carried out by Thermo Fisher Scientific according to their standard procedures.

### 3.6. Affinity Chromatography Studies

An AKTA Avant 25 chromatography system (Cytiva, Marlborough, MA, USA) equipped with conductivity, UV, and pH detectors was used to perform small-scale studies and the intermediate scale-up at room temperature. System control and data analysis were done through UNICORN^TM^ 7.6 software. Omnifit Labware Columns (Kinesis, Portland, OR, USA) or XK 16/20 (Cytiva, Marlborough, MA, USA) were packed with epoxide-activated agarose beads at a bed volume of 1 or 10 mL. The packing quality was evaluated by a pulse injection of 1 M NaCl where asymmetry factors between 1.2 and 1.6 were obtained. Columns were equilibrated with 10 column volumes (CV) of an equilibration buffer containing 50 mM HEPES, 150 mM NaCl at pH 7.5. After virus loading, the columns were washed with 5 CV of equilibration buffer, followed by elution with 3 CV of 50 mM HEPES, 150 mM NaCl, 800 mM arginine at pH 7.5. The strip step was performed with 5 CV of 50 mM sodium phosphate at pH 12. The collected samples (flow-through, elution, and strip) were stored at −80 °C for further analysis.

#### 3.6.1. Residence Time Optimization

To evaluate the impact of the residence time on VSVG-LVs recovery yield, a 1 mL column packed with the CaptureSelect™ Lenti VSV-G Affinity Matrix [74] was used. These experiments were performed at different residence times (1, 2, and 4 min). The columns were equilibrated, washed, and eluted as previously described. After, flow-through and elution fractions were collected and pooled based on the in-line UV signal at 280 nm and stored at −80 °C, for further analysis.

#### 3.6.2. Dynamic Binding Capacity Determination

To determine the dynamic binding capacity (DBC) for the CaptureSelect™ Lenti VSVG Affinity Matrix, a 1 mL column was used. Clarified VSV-LV feedstock was loaded onto the column at a flow rate of 0.5 mL/min. The total particle concentration (TP) was determined in the flow-through fractions. The DBC at 10% was calculated according to Equation (1), where C_0_ corresponds to initial virus concentration (TP/mL), V_b_ is the breakthrough volume at which the flow-through stream achieves 10% of the loaded concentration (mL), V_0_ is the void volume (mL), and CV is the total column bed volume (mL). The DBC was determined in TP per mL of resin.

(1) $${DBC}_{10\%}=\frac{C_{0}(V_{b}-V_{0})}{CV}$$

### 3.7. Analytics

#### 3.7.1. VSVG-LVs Transducing Particle Quantification

The HEK293T cells (ATCC ^®^ CTRL-3216 ^TM^) were acquired from the American Type Culture Collection (ATCC^®^, Manassas, VA, USA) and cultured in Dulbecco’s Modified Eagle’s Medium (Thermo Fisher Scientific™, Waltham, MA, USA), supplemented with 10% fetal bovine serum (Thermo Fisher Scientific™, Waltham, MA, USA), 4 mM of GlutaMAX™ (Thermo Fisher Scientific™, Waltham, MA, USA) and 1 mM sodium pyruvate (Thermo Fisher Scientific™, Waltham, MA, USA). The functional VSVG-LVs titers were evaluated by transducing HEK293T cells and assessing GFP expression by flow cytometry as described elsewhere [75]. Briefly, HEK293T cells in 24-well plates were transduced with VSVG-LVs at different dilutions in fresh DMEM with 10% (*v*/*v*) FBS and 8 µg/mL of polybrene (MilliporeSigma, Burlington, MA, USA) and incubated at 37 °C. After 2 h, 0.5 mL of fresh DMEM with 10% (*v*/*v*) FBS was added to each well, and the plates incubated at 37 °C. Infected cells were sorted by fluorescence microscopy (Leica) to select the wells with approximately 5–20% of positive GFP containing cells 48 h post-transduction. After trypsinization of the cells in those wells, the percentage of GFP positive cells was quantified by flow cytometry in the FACSCelesta™ equipment (BD Biosciences, San Jose, CA, USA). The concentration of VSVG-LVs transducing units (TU/mL) was calculated using Equation (2):

(2) $$Titer\left(\frac{TU}{mL}\right)=\frac{\%\;of\;GFP\;positive\;cells÷100}{volume\;of\;transduction}\times dilution\;factor\times number\;of\;cells\;transduced$$

#### 3.7.2. Total Particle Quantification

Total particles (TP) concentration was estimated using the ELISA kit Innotest HIV Antigen mAb (Fujirebio, Malvern, PA, USA). To determine the total amount of p24 protein, a standard curve was established using the positive control with a 150–25 pg/mL concentration range according to the manufacturer’s instructions. A ratio of 1.25 × 10^7^ TP/ng of p24 was used to calculate the total particles titer.

#### 3.7.3. Total Protein, Host Cell Protein, and DNA Quantification

Total protein was determined using the Pierce^TM^ BCA assay kit (Thermo Fisher™, Waltham, MA, USA) and total DNA was quantified with Quant-iT PicoGreen dsDNA assay kit (ThermoFisher™, Waltham, MA, USA) used according to the manufacturer’s instructions. Host cell protein was determined with an HEK 293 HCP ELISA Kit (Cygnus Technologies, Southport, NC, USA). The detection was performed using the Infinite PRO NanoQuant (Tecan, Männedorf, Switzerland) microplate multimode reader. The samples were applied at multiple dilutions.

#### 3.7.4. Transmission Electron Microscopy

The morphological analysis of VSVG-LVs was performed using transmission electron microscopy (TEM). Sample preparation consisted of a drop (5 µL) of each sample that was adsorbed onto formvar-coated 150-mesh copper grids from Veco (Science Services, Munich, Germany) for 2 min. Afterwards, the grid was washed 5 times with sterile dH_2_O, then soaked in 2% (*w*/*v*) uranyl acetate for 2 min and dried in air at room temperature. To analyze the grid, an Hitachi H-7650 120 Kv electron microscope (Hitachi High-Technologies Corporation, Tokyo, Japan) was used.

#### 3.7.5. Quality Analysis of VSVG-LVs Particles by CE-SDS with LIF Detector

VSVG-LVs characterization was performed by sodium dodecyl sulfate capillary electrophoresis (CE-SDS) combined with laser-induced fluorescence (LIF) detection. Sample preparation and labeling was performed as described elsewhere [68]. All the analyses were carried out in a CESI 8000 Plus system (SCIEX, Framingham, MA, USA) with a 488 nm laser-induced fluorescence detector module using an emission filter of 600 nm. The separation was performed using a bare fused silica capillary of 50 µm inner diameter, 30.2 cm in total length, and with 20.2 cm effective separation length (SCIEX, Framingham, MA, USA).

#### 3.7.6. Western Blot Analysis

The pattern of p24 protein was assessed by Western blot analysis. After protein denaturation, SDS-PAGE was carried out under reducing conditions in a 4–12% NuPage Bis-Tris protein gel (Thermo Fisher Scientific™, Waltham, MA, USA) and SeeBlue Plus2 Prestained Standard (Thermo Fisher Scientific™, Waltham, MA, USA) as molecular weight marker. The proteins were transferred to a PVDF membrane using the iBlot™ 2 system (Thermo Fisher Scientific™, Waltham, MA, USA). After blocking with Tris-Buffered Saline (Merck, Darmstadt, Germany) with 0.1% (*w*/*v*) Tween-20 (Merck, Kenilworth, NJ, USA) with 5% (*w*/*v*) skim milk (Merck, Kenilworth, NJ, USA) for 1 h, the membrane was incubated overnight with the anti-HIV-1 p24-mouse monoclonal antibody (Abcam, Cambridge, MA, USA). Afterward, the membranes were washed and incubated with the secondary antibody, anti-mouse IgG conjugated with alkaline phosphatase conjugate labeling (Merck, Darmstadt, Germany) for 1 h. The protein detection was performed using NBT/BCIP 1 Step (Thermo Fisher™, Waltham, MA, USA).

## 4. Conclusions

Nowadays, there is a fast-growing demand for gene therapy and cell-based products due to their tremendous potential for personalized medicine. Regarding LV-based therapies, downstream processing remains challenging due to the lack of simple and scalable purification trains. This works describes the discovery and implementation of a novel affinity matrix, based on CaptureSelect™ technology, which enables for the first time the capture of LVs pseudotyped with a VSV-G envelope. The novel adsorber achieved competitive yields and impurity clearance when compared with the available technologies. The results achieved in this work demonstrate the applicability of this new adsorber, which offers high selectivity, high purity, scalability, and gentle elution conditions. These features make the Lenti VSVG Affinity Matrix a reliable alternative to the traditional AEX chromatography. Moreover, the efficient purification and process simplification achieved can decrease time-to-market transition and open new possibilities to address the challenge of large-scale LVs purification.

## Figures and Tables

**Table 1:** Properties of the matrices explored for the establishment of a new affinity resin.

Matrix Type	Coupling Method	Selectivity Profile	Average Particle Size (μm)
Matrix 1	Direct	−	50
Matrix 2	Direct	+/−	50
Matrix 3	Direct	+++	65
Matrix 4	Biotin	++	45–165

**Table 2:** LVs quality assessment for the intermediate scale (10 mL resin) purification process using the novel Lenti VSVG Affinity Matrix. (*n* = 3).

Fraction	Transduction Units (TU/mL)	Volume (mL)	Total Particles (TP/mL)	TP/TU Ratio	Residual HCP (%)
Load	7.3 ± 2.1 × 10^7^	250	1.0 ± 1.5 × 10^10^	143 ± 23	-
Flow-through	8.1 ± 8.9 × 10^6^	258	1.7 ± 6.8 × 10^9^	270 ± 107	98.5 ± 1.3
Elution	3.7 ± 1.5 × 10^8^	22.5	3.3 ± 1.0 × 10^10^	97 ± 40	1.5 ± 1.2

## Data Availability

The sensitive nature of some of the reagents used in this study (e.g., cell lines, plasmids, baculoviruses, and antibodies) means that they are only readily available internally to the author’s institution staff for R&D purposes. For external researchers, the approval of reagent requests may be obtained via email addressed to the corresponding author.

## Supplementary Materials

The following supporting information can be downloaded at: https://www.mdpi.com/article/10.3390/ijms24043354/s1.
