# Supplementary material for: Implementation of Novel Affinity Ligand for Lentiviral Vector Purification
Source: Int J Mol Sci. 2023 Feb 8;24(4):3354. doi: 10.3390/ijms24043354 (PMC9966744; doi:10.3390/ijms24043354)
Supplement: Supplementary file 1 [file ijms-24-03354-s001.zip › ijms-2160729-supplementary.pdf]

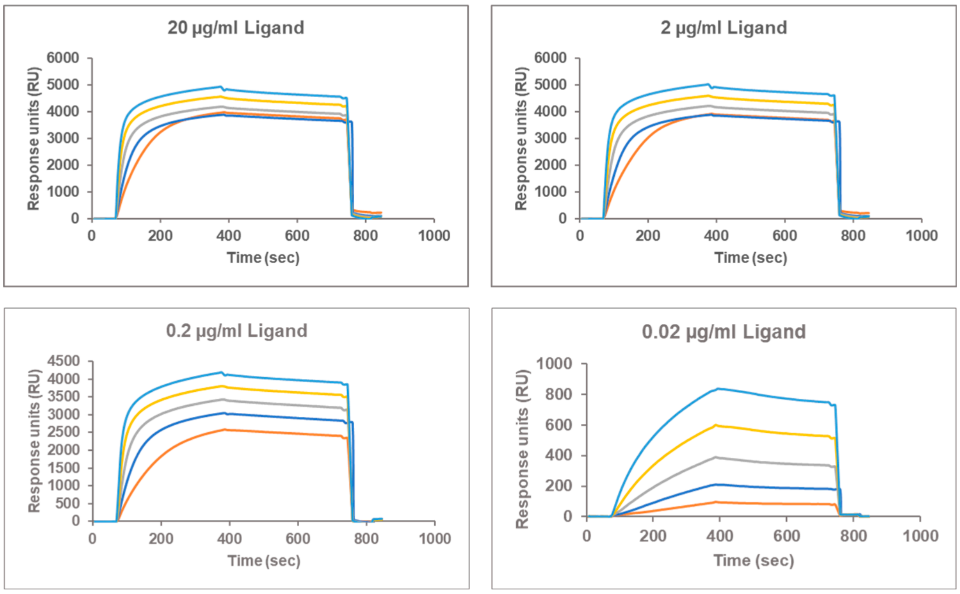

Figure S1. Representation of the SPR sensorgrams containing the relative response (RU) versus time (s) for ligand A. Different binding responses were obtained by varying the dilution of VSV-G pseudotyped LVs feedstock injected (2-fold dilutions from 10 to 160 times). Color code: blue represents 10 times dilution, green represents 20 times dilution, yellow represents 40 times dilution, red represents 80 times dilution, and gray represents 160 times dilution.

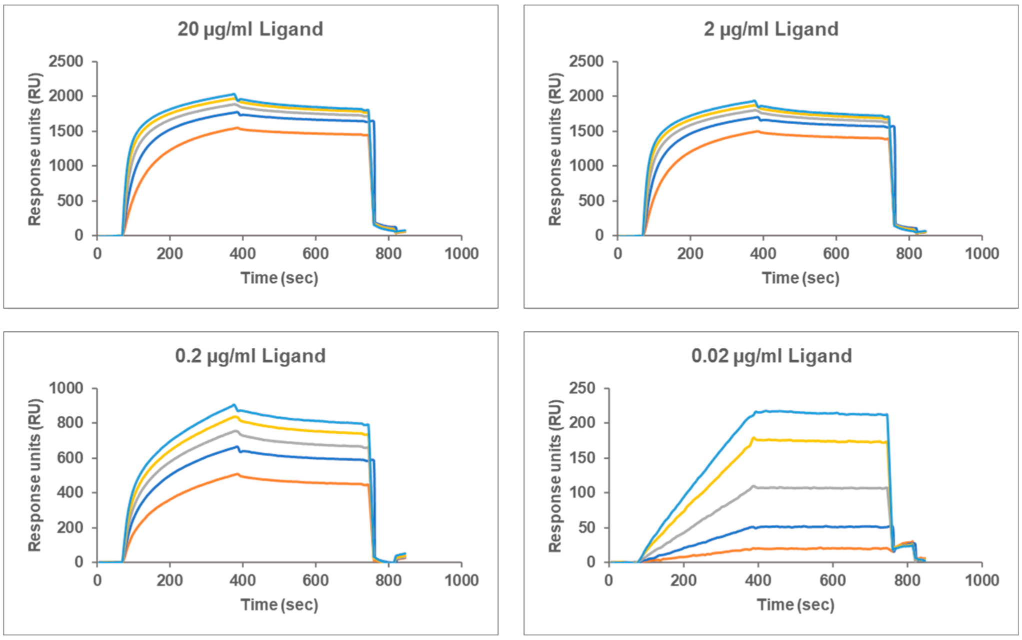

Figure S2. Representation of the SPR sensorgrams containing the relative response (RU) versus time (s) for ligand B. Different binding responses were obtained by varying the dilution of a VSV-G pseudotyped LVs feedstock injected (2-fold dilutions from 10 to 160 times). Color code: blue represents the 10 times dilution, green represents 20 times dilution, yellow represents 40 times dilution, red represents 80 times dilution, and gray represents 160 times dilution.

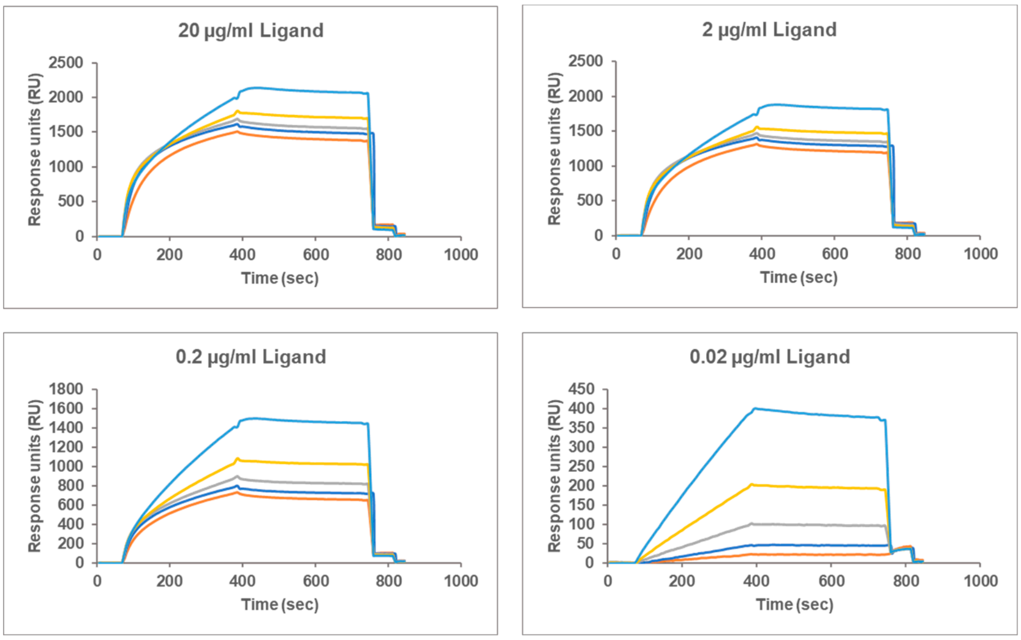

Figure S3. Representation of the SPR Sensorgrams containing the relative response (RU) versus time (s) for ligand C. Different binding responses were obtained by varying the dilution of VSV-G pseudotyped LVs feedstock injected (2- fold dilutions from 10 to 160 times). Color code: blue represents the 10 times dilution, green represents 20 times dilution, yellow represents 40 times dilution, red represents 80 times dilution, and gray represents 160 times dilution.

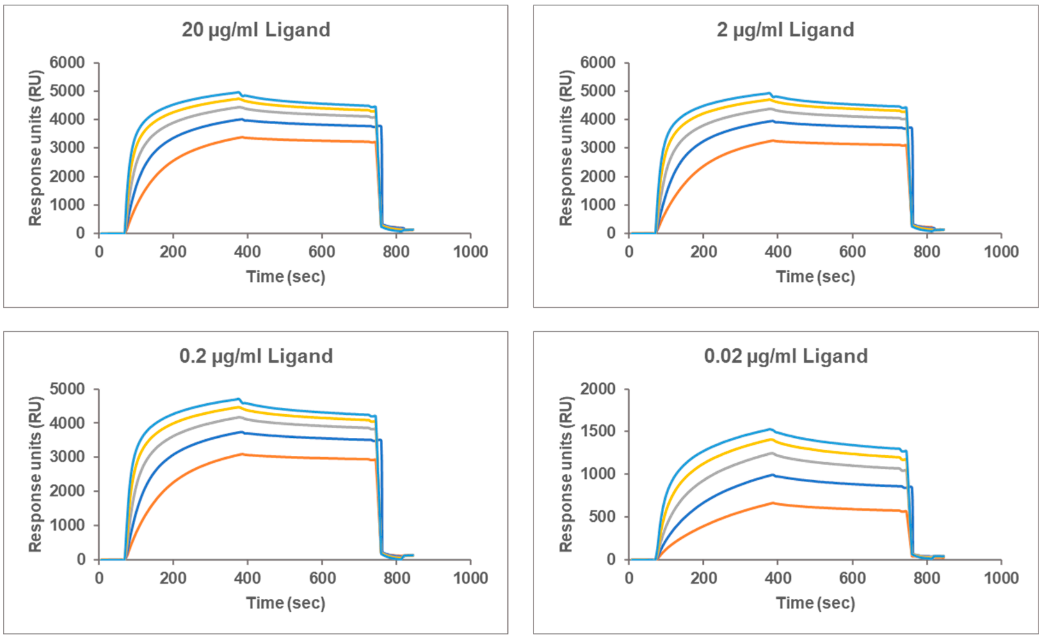

Figure S4. Representation of the SPR sensorgrams containing the relative response (RU) versus time (s) for ligand D. Different binding responses were obtained by varying the dilution of a VSV-G pseudotyped LVs feedstock injected (2- fold dilutions from 10 to 160 times). Color code: blue represents the 10 times dilution, green represents 20 times dilution, yellow represents 40 times dilution, red represents 80 times dilution, and gray represents 160 times dilution.

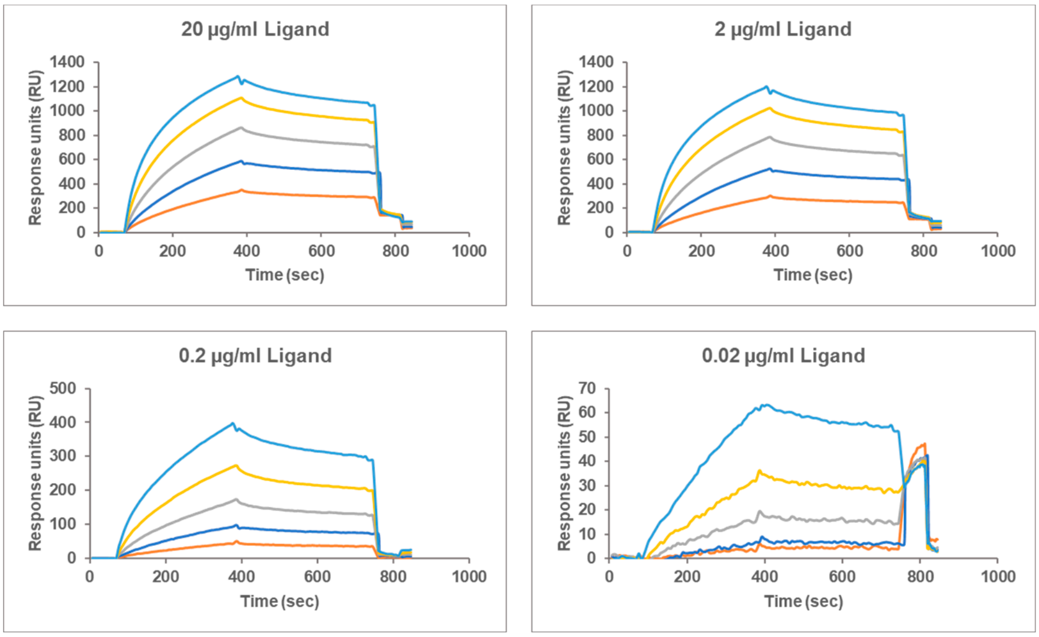

Figure S5. Representation of the SPR sensorgrams containing the relative response (RU) versus time (s) for ligand E. Different binding responses were obtained by varying the dilution of a VSV-G pseudotyped LVs feedstock injected (2-fold dilutions from 10 to 160 times). Color code: blue represents the 10 times dilution, green represents 20 times dilution, yellow represents 40 times dilution, red represents 80 times dilution, and gray represents 160 times dilution.

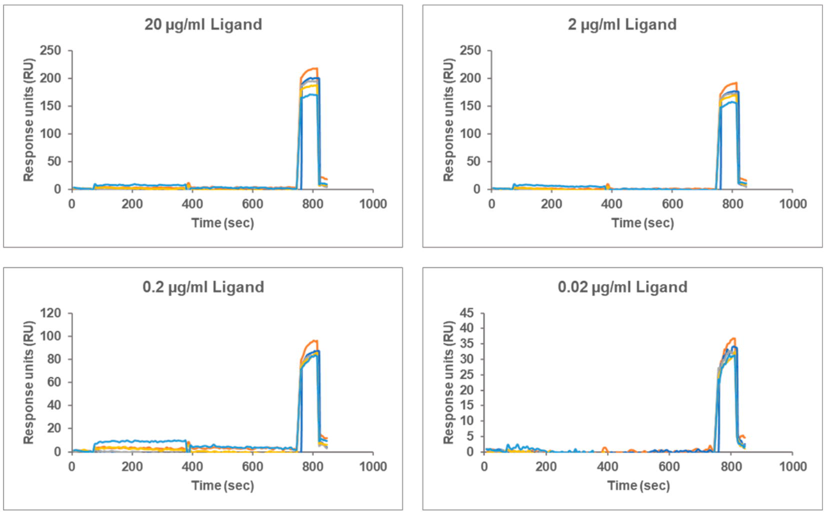

Figure S6. Representation of the SPR sensorgrams containing the relative response (RU) versus time (s) for the negative control BacuClear ligand. Different binding responses were obtained by varying the dilution of a VSV-G pseudotyped LVs feedstock injected (2-fold dilutions from 10 to 160 times). Color code: blue represents the 10 times dilution, green represents 20 times dilution, yellow represents 40 times dilution, red represents 80 times dilution, and gray represents 160 times dilution.

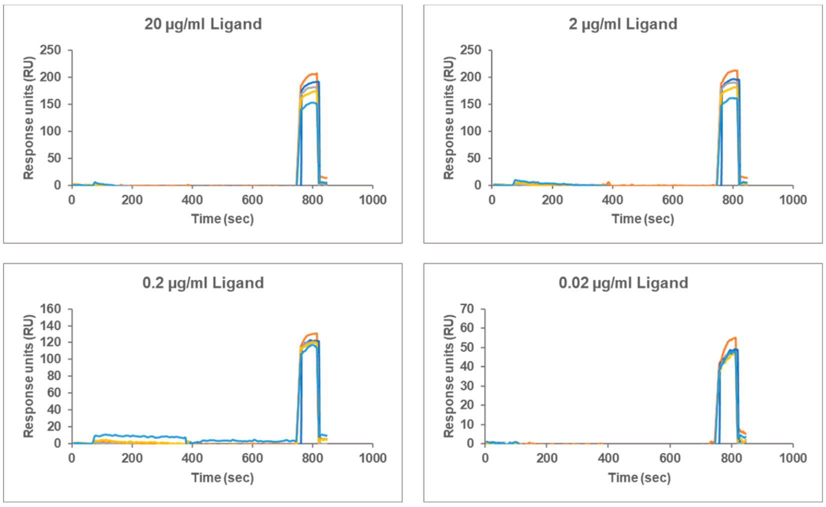

Figure S7. Representation of the SPR sensorgrams containing the relative response (RU) versus time (s) for the negative control AAVx ligand. Different binding responses were obtained by varying the dilution of VSV-G pseudotyped LVs feedstock injected (2-fold dilutions from 10 to 160 times). Color code: blue represents the 10 times dilution, green represents 20 times dilution, yellow represents 40 times dilution, red represents 80 times dilution, and gray represents 160 times dilution.
